# Individual and group-level optimization of electric field in deep brain region during multichannel transcranial electrical stimulation

**DOI:** 10.3389/fnins.2024.1332135

**Published:** 2024-03-11

**Authors:** Hidetaka Nishimoto, Sachiko Kodera, Naofumi Otsuru, Akimasa Hirata

**Affiliations:** ^1^Department of Electrical and Mechanical Engineering, Nagoya Institute of Technology, Nagoya, Japan; ^2^Center of Biomedical Physics and Information Technology, Nagoya Institute of Technology, Nagoya, Japan; ^3^Institute for Human Movement and Medical Sciences, Niigata University of Health and Welfare, Niigata, Japan; ^4^Department of Physical Therapy, Niigata University of Health and Welfare, Niigata, Japan

**Keywords:** electrode montage, individual head model, optimization, transcranial electrical stimulation, volume conductor model

## Abstract

Electrode montage optimization for transcranial electric stimulation (tES) is a challenging topic for targeting a specific brain region. Targeting the deep brain region is difficult due to tissue inhomogeneity, resulting in complex current flow. In this study, a simplified protocol for montage optimization is proposed for multichannel tES (mc-tES). The purpose of this study was to reduce the computational cost for mc-tES optimization and to evaluate the mc-tES for deep brain regions. Optimization was performed using a simplified protocol for montages under safety constraints with 20 anatomical head models. The optimization procedure is simplified using the surface EF of the deep brain target region, considering its small volume and non-concentric distribution of the electrodes. Our proposal demonstrated that the computational cost was reduced by >90%. A total of six–ten electrodes were necessary for robust EF in the target region. The optimization with surface EF is comparable to or marginally better than using conventional volumetric EF for deep brain tissues. An electrode montage with a mean injection current amplitude derived from individual analysis was demonstrated to be useful for targeting the deep region at the group level. The optimized montage and injection current were derived at the group level. Our proposal at individual and group levels showed great potential for clinical application.

## 1 Introduction

Transcranial electric stimulation (tES) (Stagg and Nitsche, 2011) and transcranial magnetic stimulation (TMS) (Kobayashi and Pascual-Leone, 2003) attract attention for its application to neuromodulation as well as neuroscience. In tES, weak current is injected through electrodes attached to the scalp, inducing the electric field (EF), which is a physical agent, in a specific region of the brain. The EF may cause neuromodulation or plasticity.

Among tES, transcranial direct current stimulation (tDCS) or transcranial alternating current stimulation (tACS) has been widely used. In tES, the target area was mainly on the shallow region of the brain, while its applications to deeper regions (Nitsche et al., 2009; Tavakoli and Yun, 2017), such as cerebrum (Ferrucci et al., 2015), subcortical, subthalamus, and hippocampus (Huang et al., 2017; Chhatbar et al., 2018; Vöröslakos et al., 2018; Gomez-Tames et al., 2019a) are suggested to be promising. tES is used as a treatment for Parkinson’s disease (Goodwill et al., 2017), depression (Nitsche et al., 2009; Palm et al., 2016), addiction (Sauvaget et al., 2015; Ekhtiari et al., 2019), anxiety (Sagliano et al., 2019), etc. Some of these diseases are reported to be associated with disorders of reward system in the brain (Berton and Nestler, 2006; Dichiara and Bassareo, 2007). The reward system is composed of a complex network among the nucleus accumbens, dorsolateral prefrontal cortex, amygdala, hippocampus, etc., (Rodman et al., 2012). Therefore, focal stimulation is needed to avoid unintentional side effects and achieve the expected therapeutic effect. Localized current on or away from specific brain regions may lead to a better understanding of the mechanisms and neural networks in general and of the observed experimental effects of tDCS (Sadleir et al., 2012).

In tES, several approaches have been conducted to evaluate the EF in the target region, including electrode montage (Bikson et al., 2010; Edwards et al., 2013; Opitz et al., 2015; Mikkonen et al., 2020; Salvador et al., 2021; Caulfield and George, 2022), multi-pair (channel) of tES electrodes (Datta et al., 2009; Khan et al., 2022), as well as uncertainty analysis (Saturnino et al., 2019b) and microscopic skin modeling (Gomez-Tames et al., 2016; Khadka and Bikson, 2020). One difficulty in the stimulation of the deep brain region is how to shape the EF in the target region because the EF is significantly affected by the head anatomy and tissue conductivity (Bragard et al., 1996; Gomez-Tames et al., 2018). Since the individualized human head modeling has become widely used, the optimization of the EF at individual level and group level using statistical analysis has been conducted (Laakso et al., 2015; Gomez-Tames et al., 2019a). Several studies conducted the optimization using multichannel tES (Khan et al., 2022; Lee et al., 2023). Multichannel tDCS was optimized with constrained maximum intensity (D-CMI) to reduce side effects and skin-layer sensations caused by current distribution for each individual (Khan et al., 2022). In the previous study (Lee et al., 2023), mean injection current optimized for different individual head models was suggested to improve focality compared with the optimization for a standard head model. For targeting the deep brain tissue, temporal interference method is also optimized with genetic algorithm (Stoupis and Samaras, 2022). A fast computational method has been proposed under slightly looser constraints with a constant return electrode for optimizing for the average field strength at the target (Saturnino et al., 2019a). In most conventional studies, the target region was mainly the motor cortex and somatosensory cortex, which are located in the shallow region of the brain. In addition, the optimization was performed on the volumetric EF over the brain, which may result in computational burden. If the computational cost can be suppressed, optimized montage can be easily explored at the individual and group levels.

The purpose of this study is to propose a fast optimization procedure and demonstrate the effectiveness of optimized electrode conditions for the deep brain region in multichannel tDCS at the individual and group levels. The feature of the deep brain region or tissue is that its volume is relatively small, and then, the EF around the region is difficult for focal stimulation (Guler et al., 2016). Considering these features, the surface EF on the target region instead of full volumetric data of EF was used for the optimization.

## 2 Methods

### 2.1 Electric field computation methods

#### 2.1.1 Head model

**Figure 1:**
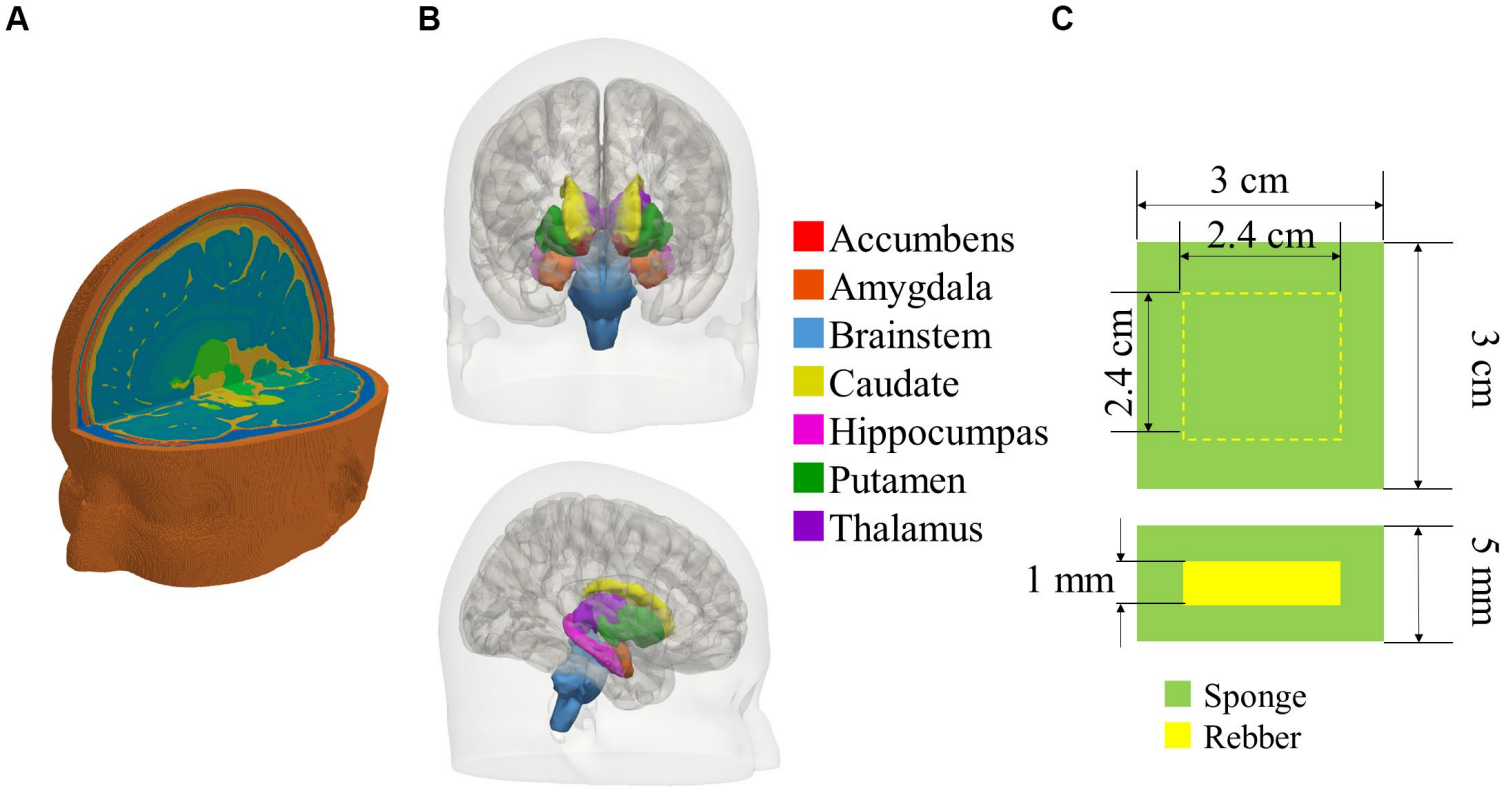
**(A)** Bird view of a human head model, **(B)** classification of deep brain tissues in head model, and **(C)** cross-sectional view of the electrode, comprised a rubber covered by a sponge.

In our previous study, 20 human head models were developed from magnetic resonance images (MRIs) (Laakso et al., 2015). The subjects were male, with a mean age of 41 ± 11 (standard deviation) years. The models had 0.5 mm resolution and were segmented into the following tissues: blood, cerebellum gray matter, cerebellum white matter, cerebrospinal fluid (CSF), cortical, dura, eyes, fat, gray matter, muscle, pallidum, deep brain regions (amygdala, nucleus accumbens, brainstem, caudate, hippocampus, putamen, and thalamus), skin, ventral diencephalon, ventricular, and white matter. The deep brain regions were defined by seven tissues located 4.5–6.5 cm deep from the scalp, involved in neuropsychiatric disorders such as depression (Levkovitz et al., 2009). An example of a human head model and the classification of deep brain tissues are shown in Figures 1A,B.

#### 2.1.2 Electrode design

The electrode is composed of a square conductive rubber sheet with a 1 mm thickness covered by a saline-soaked sponge, as shown in Figure 1C. The length of one side of the sponge is 3 cm. The position of the center of each electrode was per the 10–20 international EEG (Electro Encephalo Graphy) system (19 locations in total). For bipolar tDCS using two electrodes, the number of combinations of electrode montages is 171, equivalent to two locations selected from 19 locations. For potential combinations of the electrode position and its side length, the electrode position was not overlapped.

#### 2.1.3 Electric field computation

The human head model is set as the volume conductor. The scalar-potential finite difference method (Dawson and Stuchly, 1998) was used to calculate the scalar potential in the human head model.


∇(σ∇φ)=0

where σ and φ denote the tissue conductivity and scalar potential.

The scalar potential is defined as an unknown parameter at each node of each voxel, and the conductance is assigned to the edges with tissue conductivity. Given the simultaneous equations for current based on Kirchhoff’s current law at all nodes, we solved the scalar potential using the multigrid method with successive over-relaxation method (Laakso and Hirata, 2012). The number of multigrid level was six, and the calculation was continued until the relative residual <10^−6^ (Laakso and Hirata, 2012).

The head model EF was calculated by dividing the potential difference between adjacent voxels by the node distance. As postprocessing (Gomez-Tames et al., 2017), the top 0.1th percentile value of EF was removed (99.9th percentile) to exclude potential numerical artifact and replace with the 99.9th (Reilly and Hirata, 2016). The electrical conductivity of the tissue in the head model was assigned on the basis of the measured values to calculate EF, as conductivity of each tissue is linear and isotropic (Table 1; Laakso et al., 2015). The measured gray matter conductivity was reported to be 0.1–0.3 S/m (Freygang and Landau, 1955; Ranck, 1963; Stoy et al., 1982; Tay et al., 1989; Gabriel et al., 1996; Latikka et al., 2001; Akhtari et al., 2006, 2010), and the gray matter conductivity was 0.2 S/m. In addition, the white matter conductivity was set to 70% of the gray matter conductivity (Freygang and Landau, 1955; Stoy et al., 1982; Gabriel et al., 1996). The conductivity of the other tissues was set as follows: 0.2 S/m blood (Gabriel et al., 1996); 0.008 and 0.027 S/m compact and spongy bone (Akhtari et al., 2002); 1.8 S/m CSF (Baumann et al., 1997); 0.16, 0.08, and 0.08 S/m muscle, skin, and fat (Gabriel et al., 2009); 1.5 S/m eye humor (Lindenblatt and Silny, 2001); 0.16 S/m dura, same as muscle, optional.

**Table 1:** Electric conductivity of tissues in head model.

Tissue	Conductivity [S/m]
Amygdala	0.2
Blood	0.7
Bone (cancellous)	0.027
Bone (cortical)	0.008
Brainstem	0.14
Caudate	0.2
Cerebellum gray matter	0.2
Cerebellum white matter	0.2
CSF	1.8
Fat	0.08
Gray matter	0.2
Hippocampus	0.2
Intervertebral disk	0.1
Muscle	0.16
Nucleus accumbens	0.2
Putamen	0.2
Skin	0.1
Thalamus	0.2
Vitreous humor	1.5
White matter	0.14

### 2.2 Optimization procedure

**Figure 2:**
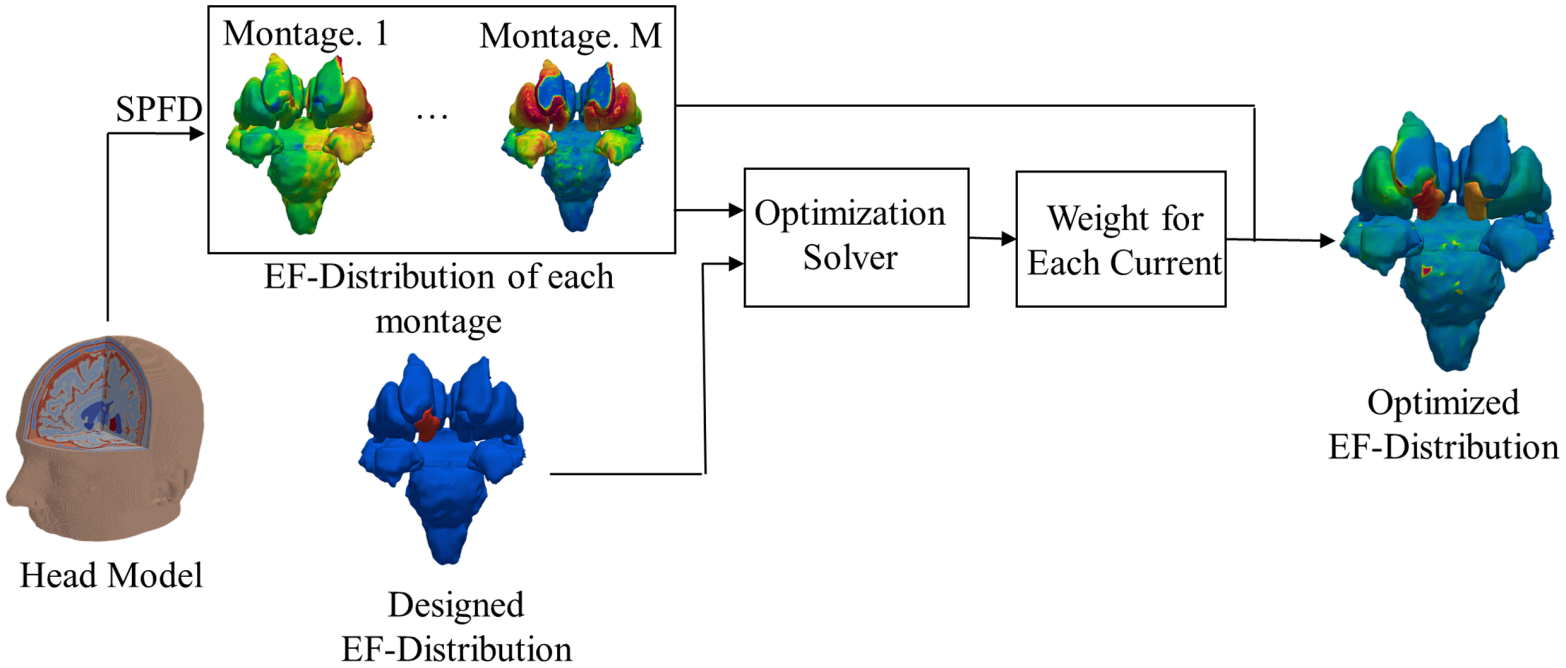
Flowchart of electromagnetic field computation and optimized EF; for clarity, EF around the target area is shown without the remaining brain.

A ratio of the optimized injection current was calculated for each montage by matching the computed and targeted EFs in the deep regions. The optimization of EFs is shown in Figure 2.

The optimized EF distribution for electrodes with the number of *k* was computed as follows: first, *E_Dataset_*, an array consisting of EF components in deep brain regions for all montages *M*, was calculated. A dataset of the EF surface in the deep brain region was also considered. *M* is defined as the number of combinations to select two electrodes from *k* electrode position, calculated for *M = _k_*C_2_. *E_Desinged_* is the EF component in the target deep brain region to be generated by externally injecting multicurrents. Notably, only EF in the deep brain region is considered at this stage.

Next, for these two input datasets, the weighing coefficient of current was computed using an optimization solver. Particularly, an array of the optimal weighting coefficient for *E_Dataset_* was calculated for _19_*C_k_* cases, the number of combinations to select the electrode position *k* from the international 10–20 system (19 locations excluding the ground). For all patterns, the root mean square error (RMSE) between *E_Desinged_* and the optimized EF was calculated. Finally, the pattern for minimal RMSE was derived for electrodes with the number of *k* as an optimized result, except in cases where the magnitude of the injected current for each electrode exceeds 4 mA to avoid skin burning or tolerability.

#### 2.2.1 Computation of dataset

*E_Dataset_* was computed for optimization:


$$\begin{array}{l} E_{Dataset}=\left[E_{1}\;\;\cdots \;\;E_{m}\right]=\left[\begin{array}{ccc} E_{1}\left(r_{1}\right) & \ldots & E_{m}\left(r_{1}\right)\\ \vdots & \ddots & \vdots \\ E_{1}\left(r_{N}\right) & \ldots & E_{m}\left(r_{N}\right) \end{array}\right]\\ \;\;\;\;\;\;\;\;\;\;\;\;\;\;\;\;\;\;\;\;=\left[\begin{array}{c} \begin{array}{ccc} E_{x1}\left(r_{1}\right) & \cdots & E_{xm}\left(r_{1}\right)\\ E_{y1}\left(r_{1}\right) & \cdots & E_{ym}\left(r_{1}\right)\\ E_{z1}\left(r_{1}\right) & \cdots & E_{zm}\left(r_{1}\right) \end{array}\\ \begin{array}{ccc} \vdots & \ddots & \vdots \end{array}\\ \begin{array}{ccc} E_{x1}\left(r_{N}\right) & \cdots & E_{xm}\left(r_{N}\right)\\ E_{y1}\left(r_{N}\right) & \cdots & E_{ym}\left(r_{N}\right)\\ E_{z1}\left(r_{N}\right) & \cdots & E_{zm}\left(r_{N}\right) \end{array} \end{array}\right], \end{array}$$

where *N* is the number of voxels or elements for volumetric and surface data in the deep brain region, *m* is the number of combinations to select two electrodes from *k* electrode positions with maximum and minimum values of 171 and 1, respectively, and *E*_m_(*r*_1_) is the EF vector at location *r*_1_ induced for the montage *m*.

EFs used for constructing *E_Dataset_* are based on three Cartesian field components. The volumetric data at 0.5 mm in the deep brain region have a large number of elements (Table 2). In this study, we considered two *E_Dataset_*, using computed EF vectors over volume and only surface EF vectors on the deep brain region. The advantages of using surface data would be expected especially when optimizing head models with high resolution. If the model resolution was increased, computational cost with the number of elements in a voxel cubic model would increase cubically whereas squared for surface data.

**Table 2:** Comparison of volumetric voxel data with surface data; mean computational size for 20 subjects.

Type	Volumetric voxel data	Surface data
Number of elements in deep brain region	$\left(5.2\pm 0.46\right)\times 10^{5}$	$\left(2.3\pm 0.020\right)\times 10^{4}$
Number of elements in right accumbens	$\left(1.4\pm 0.26\right)\times 10^{4}$	$\left(1.9\pm 0.22\right)\times 10^{3}$
Number of elements in right amygdala	$\left(4.2\pm 0.42\right)\times 10^{4}$	$\left(3.7\pm 0.20\right)\times 10^{3}$
Number of elements in right putamen	$\left(1.3\pm 0.18\right)\times 10^{5}$	$\left(6.0\pm 0.14\right)\times 10^{3}$
File size of EF per montage	2.0±0.18 [MB]	12.3±1.08 [KB]

The procedure for registering EF surfaces (calculated at 1 mm depth within 7-mm-deep brain regions) on the surface representation of an individual’s brain region defined using FreeSurfer (Dale et al., 1999) was similar to cerebellar registration in our previous study (Gomez-Tames et al., 2019b).

The comparison of computational resources needed to generate volumetric voxel and surface data (including file output) is summarized in Table 2. The workstation used in this study is 16 Intel® Xeon®CPUs running at 3.4 GHz, with 256 GB memory and NVIDIA RTX A2000 GPUs. Surface data are lighter than voxel data, so the use of surface data can reduce computational resources for optimization (Table 2).

#### 2.2.2 Target tissue

**Figure 3:**
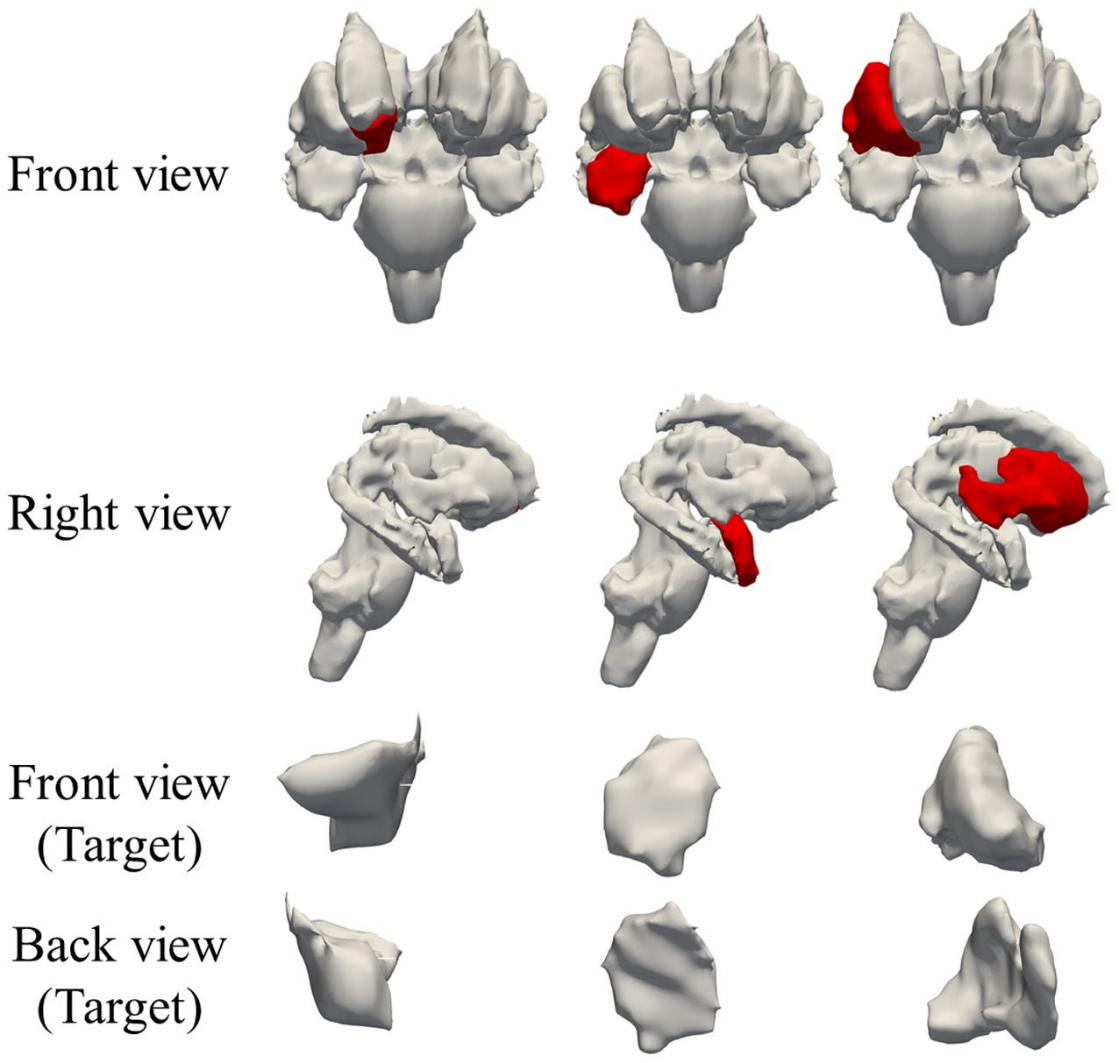
Regions targeted by electric field optimization in deep brain region: right accumbens, right amygdala, and right putamen; target regions are marked in red.

Three tissues related to mood disorders and Parkinson’s disease, namely, right side of accumbens, amygdala, and putamen, were selected as optimization targets in the deep brain region (Huff et al., 2010; Montgomery et al., 2011; Langevin et al., 2016). The input array *E_Desinged_* for the optimization equation is shown in Figure 3 and defined as follows:

$E_{Designed}=\left[\begin{array}{c} e_{d}\left(r_{1}\right)\\ \vdots \\ e_{d}\left(r_{n}\right)\\ \vdots \\ e_{d}\left(r_{N}\right) \end{array}\right]$ , $\;e_{d}\left(r_{n}\right)=\{\begin{array}{c} \begin{array}{cc} e_{0} & n\in T \end{array}\\ \begin{array}{cc} 0 & n\in T^{c} \end{array} \end{array}$ where *N* is the number of voxels or elements for volumetric and surface data in the deep brain region, *e*_d_(*r_n_*) is EF vector at location *r_n_*, *e_0_* is the EF vector designed in the target region, *T* is a set of voxels in the target region, and *T^c^* is a set of voxels in the non-target region. The amplitude of *e_0_* is 1, and the direction is horizontal.

#### 2.2.3 Constrained least squares

**Figure 4:**
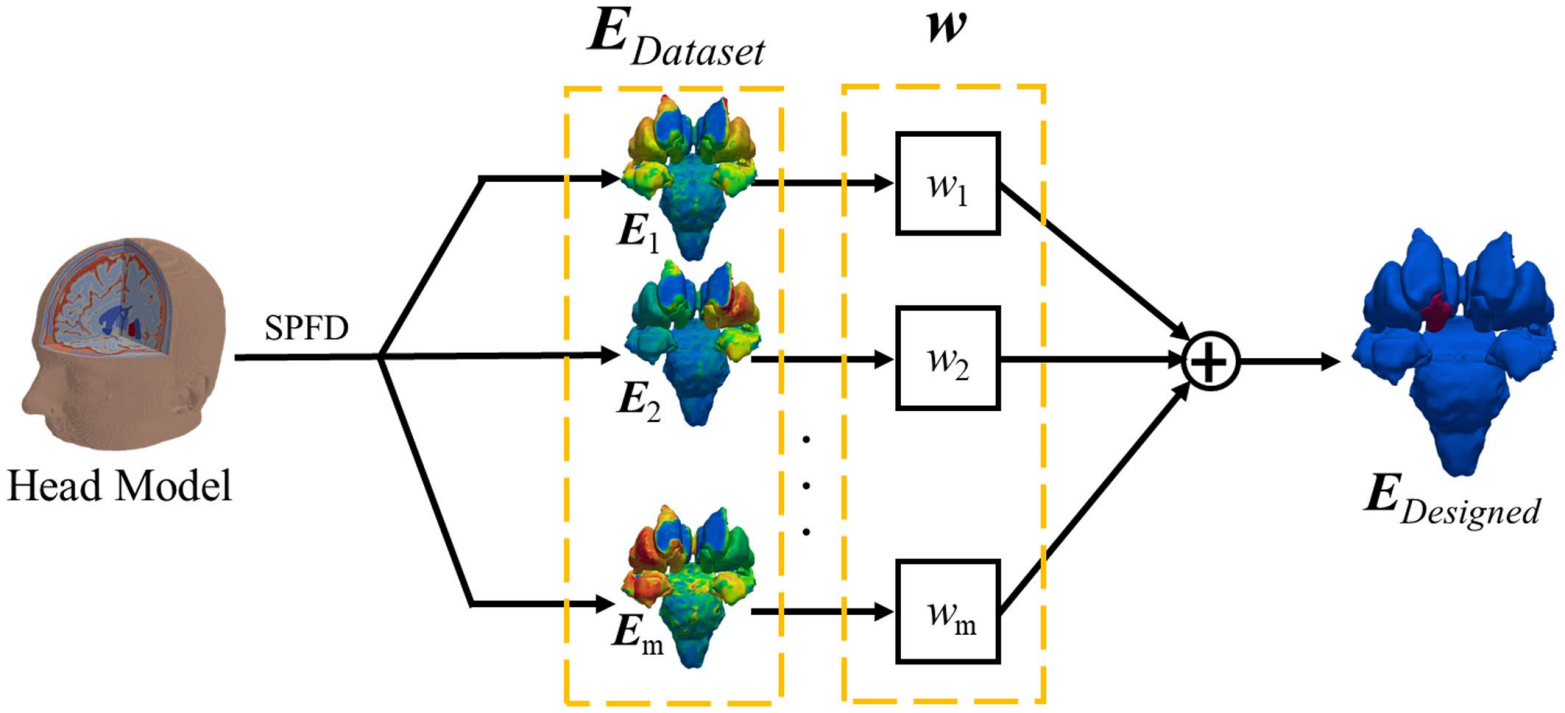
Optimum electric field calculation by linear addition of several designed electric fields.

We aimed to conduct focal stimulation by combining EFs induced by different montages (Figure 4). This problem can be described as follows:


$$\begin{array}{l} E_{1}\times w_{1}+E_{2}\times w_{2}+\ldots +E_{m}\times w_{m}=\left[\begin{array}{ccc} E_{1} & \cdots & E_{m} \end{array}\right].\left[\begin{array}{c} w_{1}\\ \vdots \\ w_{m} \end{array}\right]\\ \;\;\;\;\;\;\;\;\;\;\;\;\;\;\;\;\;\;\;\;\;\;\;\;\;\;\;\;=E_{Dataset}.w=E_{Designed} \end{array}$$

where *m* is the number of montages, and *w* is the current ratio for each montage.

The current induced in the brain by tDCS is more spread out in deeper regions of the brain (Gomez-Tames et al., 2019a), so the injected current is not concentrated in some electrodes. Therefore, the unknown *w* can be solved using the constrained least square method to avoid focusing the current at one electrode (Guler et al., 2016). We imposed the upper limit of the injection current, so each injected current from one electrode does not exceed two times that of the original:


$$Restricted:\left|w_{m}\right|\leq 2$$

The computational algorithm used the interior point method, performed using MATLAB (R2022b, MathWorks (R)).

### 2.3 EF analysis at group level

In evaluating the optimization at group level, EF at the surface of the deep brain region was projected from an individual model to the template. Briefly, each tissue surface of the deep brain region was automatically registered using affine transformation to the deep brain template. The iterative closest point transform, part of the Visualization Toolkit, was used for the registration. For each point of the template surface Y, the closest point x of the individual surface X, affine transformed, was determined by the minimum Euclidean distance (f: Y → X).

The group-level EF was defined as EF normalized by the maximum EF strength (EF absolute value) of the individual brain surface in the deep brain region, projected onto the template brain, further averaged over all subjects.

## 3 Results

### 3.1 Computational costs

**Figure 5:**
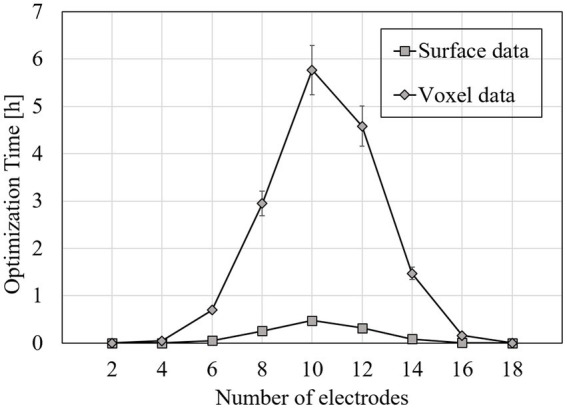
Average time required for optimization while targeting the right accumbens in one subject; optimization time was defined as computational time required to solve optimization equation for all patterns of 2–18 electrodes in one subject.

Figure 5 shows the average time required for optimization with *k* electrodes when targeting the right accumbens for all subjects. The optimization time using *k* electrodes was defined as the time required to solve the optimization equation *E*_Dataset_*w* = *E*_Designed_ of optimize the current ratio *w* for all patterns with *k* electrodes. While using the workstation, the computational cost was reduced dramatically when the EF surface data were used instead of the volumetric voxel data. The computational time and memory usage of *E*_Dataset_ were reduced by 91 and 97%, respectively, while optimizing with 19 electrodes as compared with the volumetric voxel data (Figure 5).

### 3.2 Number of electrodes

**Figure 6:**
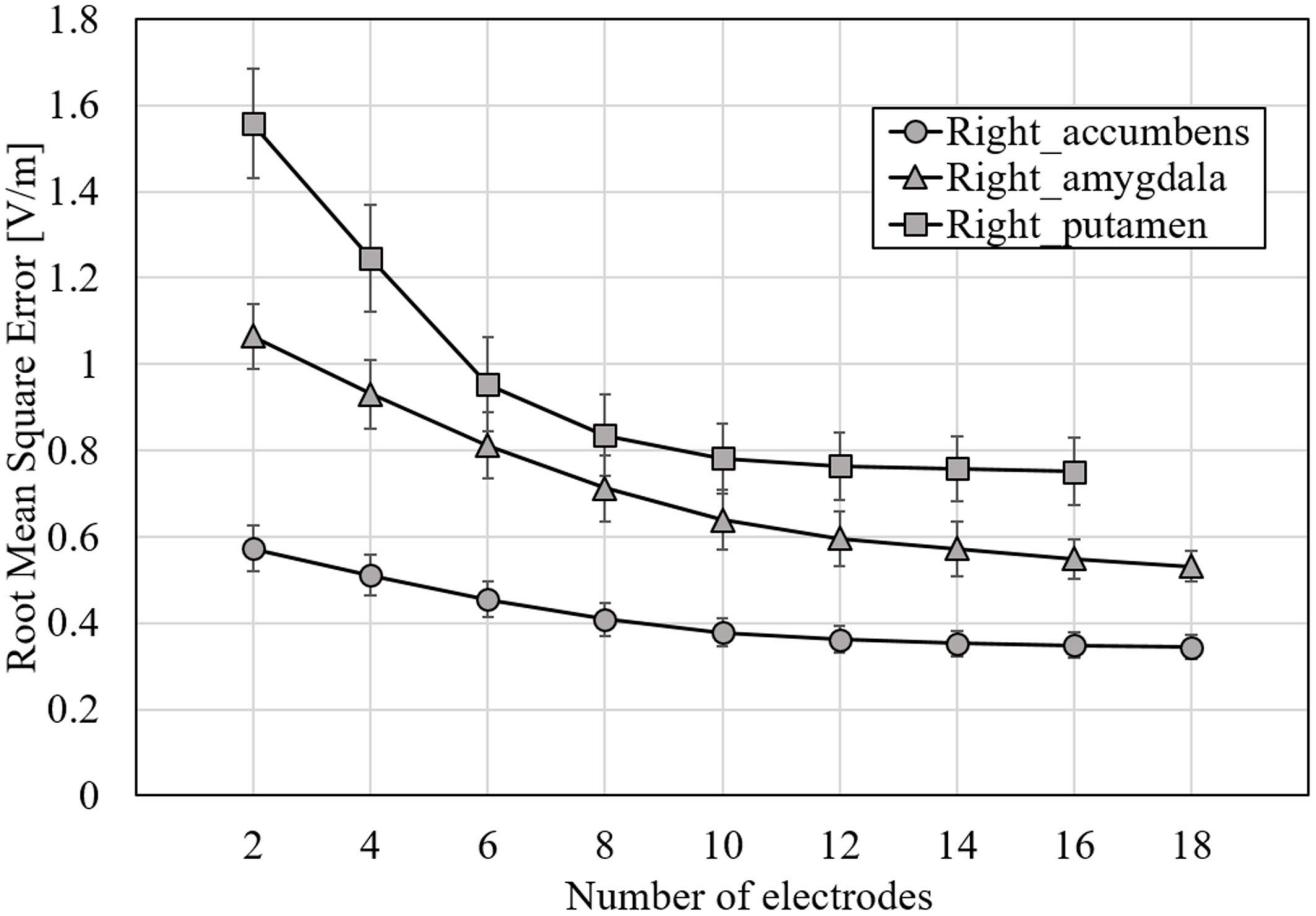
RMSE mean for optimized EF using surface data and *E*_Designed_ for all subjects; target tissues were right accumbens, right amygdala, and right putamen; errors are shown as standard deviations.

**Figure 7:**
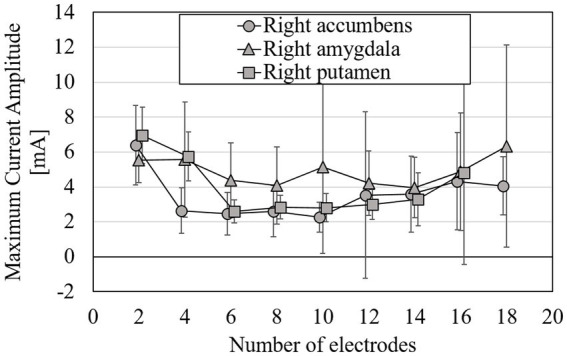
Mean of maximum intensity of injection current needed for volume-averaged EF strength of 0.4 V/m with surface data; target tissues were right accumbens, right amygdala, and right putamen; errors are shown as standard deviations.

**Figure 8:**
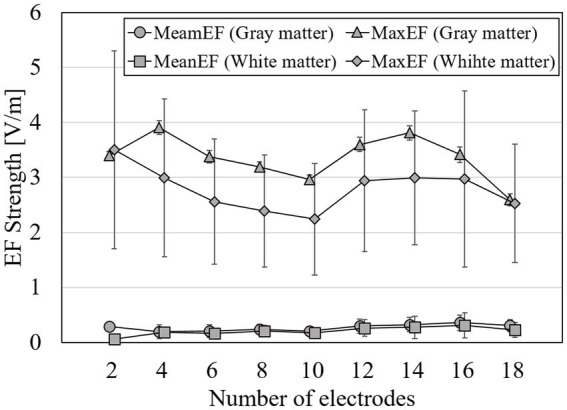
Volume-averaged and maximum EF strength in gray matter when optimization using the surface data and a total current is <4 mA at group level; error bars represent for standard deviations.

The EF strength distribution and currents were normalized so that the volumetric average EF strength in the target was 0.4 V/m, which is an estimated stimulation threshold in an EF modeling study (Laakso et al., 2019). In this subsection, results are shown using the surface data for simplicity and to avoid repetition as well as huge computational cost in volumetric approach. Figure 6 shows the RMSE mean while optimizing EF of 2–18 electrodes in one of the following target regions: right accumbens, amygdala, or putamen. Figure 7 shows the maximum current amplitude current injected into each electrode to induce the volume average of EF strength of 0.4 V/m in the target region: right accumbens, amygdala, or putamen for all subjects. To discuss the induced EF in the region outside the target region, Figure 8 shows the mean and maximum EF strength in the gray matter and white matter when optimizing the right accumbens as a target.

From Figure 6, RMSE decreases with increasing number of electrodes. The rate of change in RMSE became stable for the electrode number of about 8, regardless of the target tissue. When targeting the right putamen, RMSE decreased by 44 and 8% for 2–8 and 8–18 electrodes, respectively. When targeting the right accumbens, RMSE decreased by 11%, even for electrodes >9. The maximum current amplitude to induce the volume average of EF strength of 0.4 V/m did not exceed 4 mA at 4–14 electrodes when targeting the right accumbens; at 6–14 electrodes when the right putamen (Figure 7). When targeting the right amygdala, a current of >4 mA was required to obtain the average EF strength of 0.4 V/m for any number of electrodes.

From Figure 8, the average strength of EF in the gray matter and white matter was stable regardless of the number of electrodes, with values of 0.28 V/m both for gray matter and white matter. The maximum strength in the gray matter or white matter was the local minimum values (a value of the gray matter: 2.97 ± 1.74 V/m; the white matter: 2.45 ± 1.01 V/m) at 10 electrodes. The variation (SD) of the maximum strength in the white matter was 1.5 times higher than the variation of the gray matter.

### 3.3 Optimized EF in deep brain regions at group level

**Figure 9:**
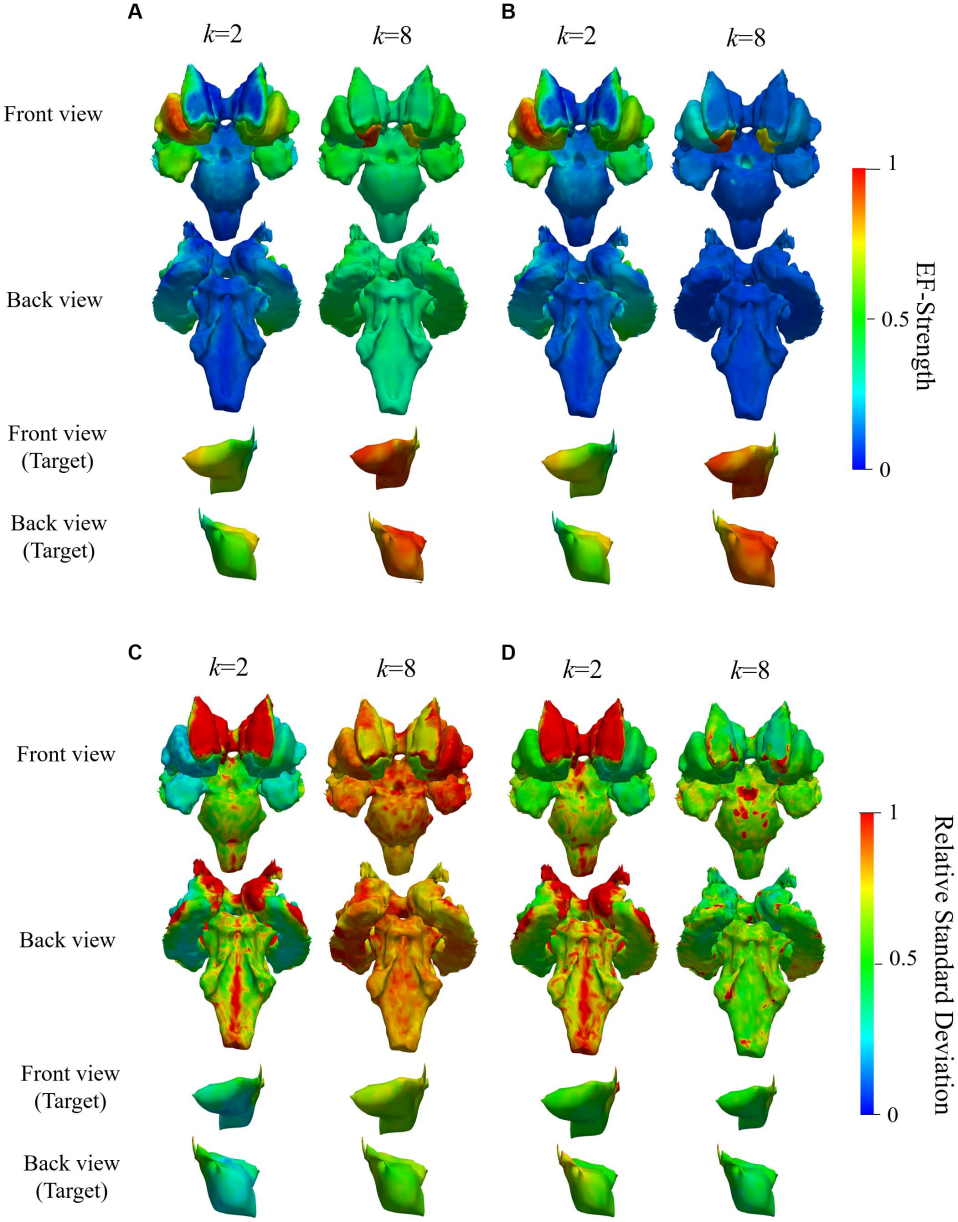
Mean and SD of normalized EF while targeting right accumbens in the deep brain region of 20 subjects; **(A,C)** are mean and relative SD of EF strength optimized using volumetric voxel data, while **(B,D)** correspond to that using surface data; figures in the bottom two rows are zoomed views of target regions; color scale is truncated at 99th percentile of corresponding EF.

**Figure 10:**
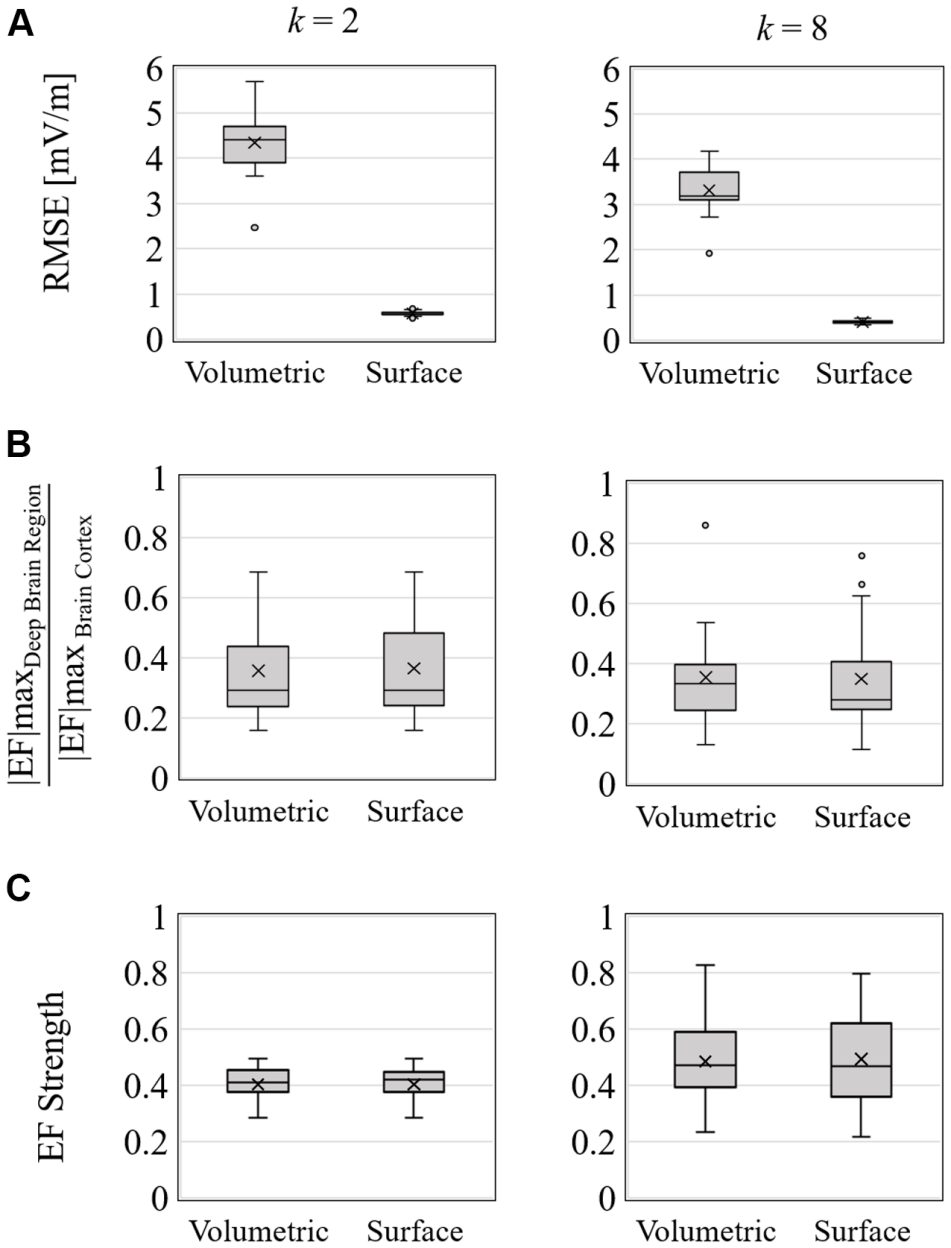
Optimization for 2 and 8 electrodes; box charts of **(A)** EF optimized RMSE, **(B)** ratio of maximum EF in deep brain region to brain cortex, and **(C)** volume average of EF strength in the target region of right accumbens in 20 subjects.

Figure 9 shows the group-level EF strength and relative standard deviations in the deep brain region while optimizing EF in the right accumbens (target region) with two and eight electrodes. Figure 10 shows box charts of the volume average EF strength in the target, its RMSE from *E*_Designed_ in deep brain regions and ratio maximum strength of EF in the deep brain region to the outer region of deep brain region.

Based on EF optimization using the EF surface, the computed induced EF in the target region was comparable to or higher than volumetric voxel data. EF in the non-target region shows low EF strength (Figures 9A,B). The EF strength variation in target tissues was insignificantly different regardless of the dataset type. In the non-target regions, regardless of the data used, lower SD was distributed in target tissue proximities, anterior caudate, and left accumbens (Figures 9C,D).

The mean RMSE decreased with increasing number of electrodes, whereas the mean EF was higher for *k* = 8 than for *k* = 2 but with larger variability (Figures 9A,C). This result was consistent regardless of the input data type. The ratio of maximum EF in the deep brain region to brain cortex was similar to the volumetric voxel data and surface data (Figure 10B), regardless of the number of electrodes. However, the interquartile range of each box chart is comparable to or smaller when using surface data than voxel data.

### 3.4 Electric field in individual head models

**Figure 11:**
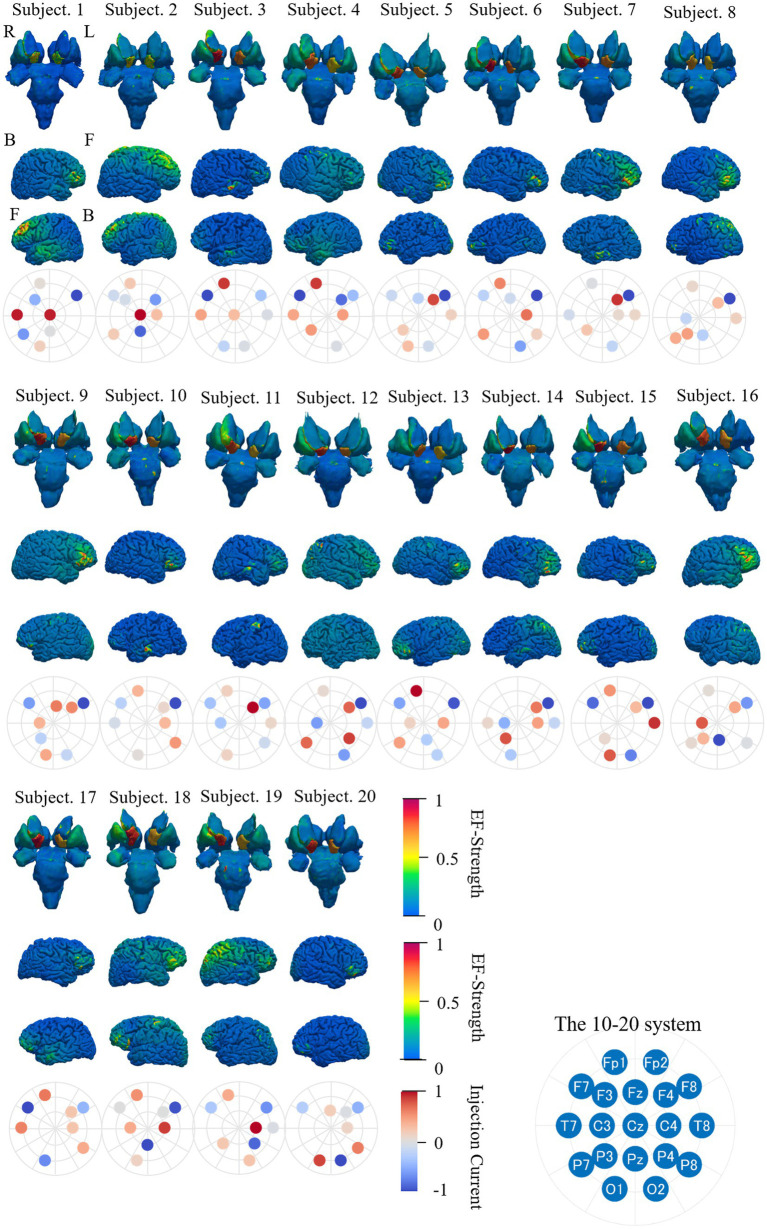
EF strength distribution in individual deep brain region, brain cortex, and electrode pattern (international 10–20 system) during optimization targeting right accumbens with eight electrodes; color scale is truncated at the 99th percentile of the corresponding EF. The electrode position with the highest injection current is circled in red. The subscripts in the figure are defined as follows: R, right side; L, left side; F, front side; B, back side.

Figure 11 shows the individual distribution of EF strength in the deep brain and cerebral regions and the selected electrode positions while optimizing EF targeting right accumbens using the surface data with eight electrodes. The EF distribution in the deep brain region and brain cortex was normalized by their respective maximums.

A higher EF is observed in the targeted region (right accumbens) than non-targeted regions for most subjects. In the majority of subjects, a higher EF was distributed in the dorsolateral prefrontal cortex, inferior frontal cortex, or region near the longitudinal fissure, depending on the montages. The location of higher EF in the cortex region was close to electrodes (F7, O2, Fz, and Pz), where the current was mostly concentrated in four electrodes. The four electrode positions, namely, F4, F7, F8, and Fp1, were selected in most subjects. The optimized electrode pattern (Cz-F7-F8-Fp1-O1-O2-T7-T8) was the same in two subjects (Subjects 10 and 17).

### 3.5 Group-level EF using the same montages

**Figure 12:**
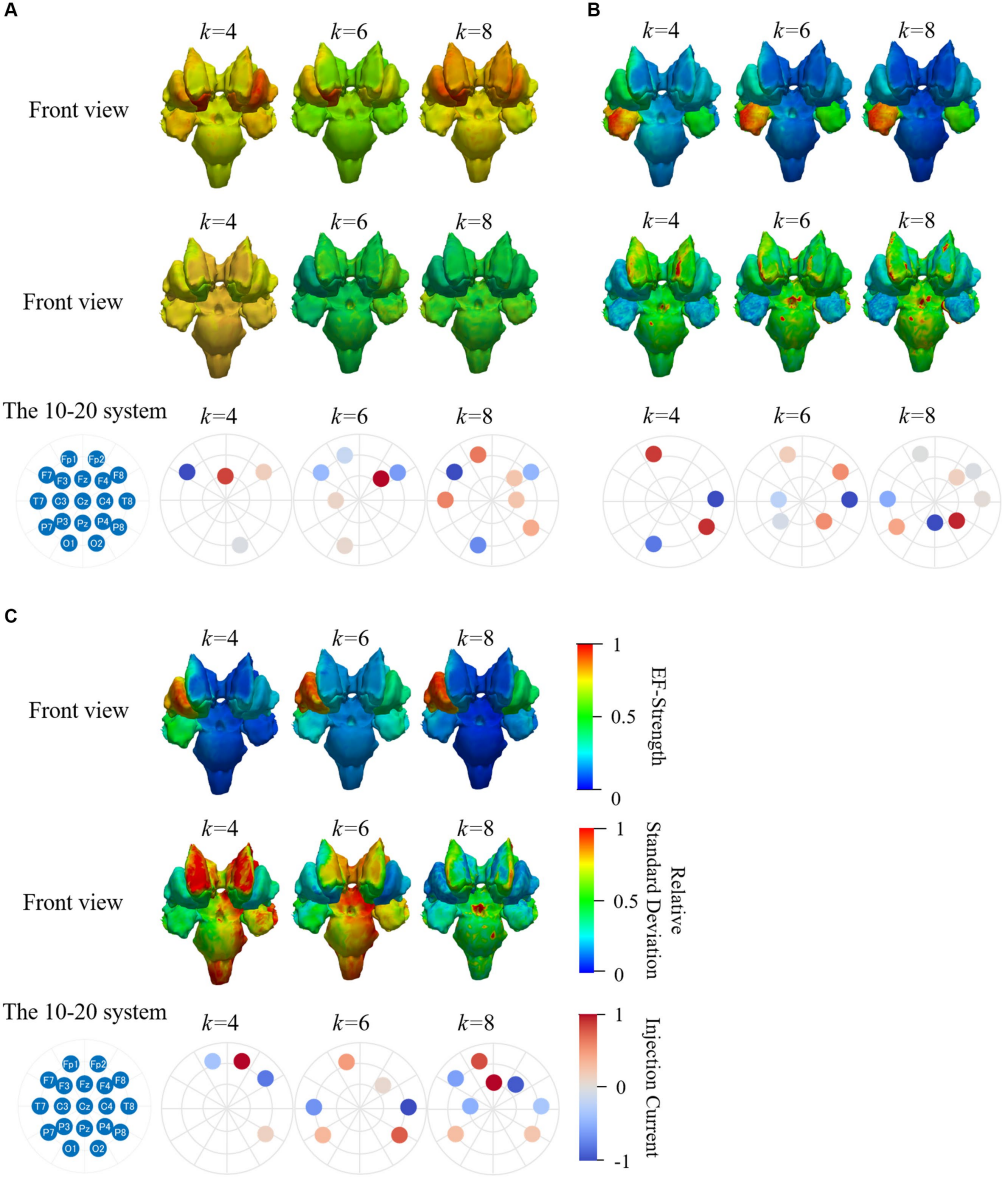
Mean and relative SD of EF strength for 20 subjects while using the same electrode pattern and the mean injection currents for targeting **(A)** right accumbens, **(B)** right amygdala and **(C)** right putamen. Color scale is truncated at 99th percentile of corresponding EF.

For potential avoidance to optimize the montage for each individual, the induced EF in the brain for group-level optimized montage was assessed. Figure 12 shows the average and relative SD of EF strength while using the same electrode pattern for all subjects and mean of optimized current ratios for each subject (Lee et al., 2023). When targeting the right accumbens (Figure 12A), the electrode montage was frequently selected for group level, F3-F8 at two electrodes (10/20 subjects), F7-F8-Fz-O1 at four electrodes (5 subjects), F7-F8-Fp1-O2-T7-T8 at six electrodes (3 subjects), and Cz-F7-F8-Fp1-O1-O2-T7-T8 at eight electrodes (2 subjects).

The frequently selected montage for group level was Fp2-T8 (15 subjects), Fp1-P3-P8-T8 (six subjects), C3-F8-Fp1-P3-P4-T8 (two subjects), and C4-Cz-F7-F8-Fp1-P3-Pz-T8 (two subjects) when targeting the right amygdala; C4-Cz (nine subjects), Fp1-Fp2-P8-T8 (five subjects), F4-F7-Fp1-P7-P8-T8 (nine subjects), and C3-C4-Cz-F7-Fp1-P7-P8-T8 (two subjects) when targeting the right putamen (Figures 12B,C).

When the same electrode position and mean injection current ratios were used, higher EFs were identified in the target area than remaining regions (Figure 12). Figure 11 shows the high variability of EF strength in the caudate nucleus, where the anatomical variability among subjects was high. The EF strength variation in other regions, including the target tissue, was insignificantly different. These results were confirmed when the number of electrodes were four, six, and eight; however, such results were not shown here to avoid repetition.

The mean distributions of field strength and relative SD obtained by optimization using the same and different eight electrode patterns for all subjects were similar (Figures 9B,C, 12). This finding suggests that this and other electrode patterns with eight electrodes may be an appropriate electrode pattern at group level.

## 4 Discussion

Several studies have been conducted for EF optimization in multichannel tDCS. In previous studies, the target area was primarily in the shallow region of the brain. tDCS-induced currents in deep brain regions are reportedly more spread out than in shallow regions (Gomez-Tames et al., 2019a). Guler et al. reported that targeting the deep region and computational cost for several configurations in the current sources is difficult (Guler et al., 2016). In previous studies of optimization for multichannel tDCS (Guler et al., 2016; Khan et al., 2022; Lee et al., 2023), the full EF volumetric data (or current density) distribution was used as an optimization input. Instead, a simpler constraint was considered but a constant return electrode was assumed in the optimization of SimNIBS (Saturnino et al., 2019a, 2021). Considering this weakness, we proposed the EF surface application over the target deep brain region as an optimization input to reduce the computational cost and for simplicity under a looser constraint but the return electrode was flexible.

The mean time required for optimization while targeting the right accumbens was reduced by >90% for the montage with 19 electrodes while using the EF surface in the target region rather than volume (Figure 5). This tendency was notable especially when the electrode number 7 to 15. Notably, the D-CMI computational time (Guler et al., 2016) is almost similar to the volumetric data in this study yet somehow smaller because the current restriction per electrode is not subjected. This finding is because the distance between the electrodes is not so close for brain stimulation; thus, the EF concentration around specific electrodes may not occur.

The RMSE mean while optimizing EF for 2–18 electrodes was used when targeting different deep brain regions. Expectedly, RMSE decreased with the increasing number of electrodes (Figure 6). However, the RMSE decrease becomes mild for >8 electrodes in different target areas. While targeting the right accumbens and putamen, a minimum of four and six electrodes were needed for the average strength of 0.4 V/m in the target under a constraint of a limit of 4 mA maximum current intensity per electrode.

When targeting the right accumbens, the EF in the outer deep brain region, gray matter, and white matter had similar average strength. However, the maximum EF strength in the outer region was >2 V/m, and the variability of the maximum strength in the white matter was higher than that in gray matter. When optimizing for the deep brain regions, it may be necessary to additionally consider tolerance in remaining regions, such as the brain cortex, in the optimization conditions.

Based on the computational results (Figure 5), EF in the target region using the EF surface data was comparable to or higher than the volumetric (voxel) data. EF in the non-target region had lower EF strength than the target region (Figures 9A,B), whereas focality in the right accumbens was not feasible. The EF strength variation in target tissues was insignificantly different regardless of the dataset type. These results indicated that the EF surface data usage is computationally efficient and useful to optimize EF in the deep brain target region. The difference in studies targeting the shallow region is that the target volume of the deep brain tissue is small, and the current is continuously governed by the continuity equation. Thus, the surface data would be enough for optimization purposes. High EF in the target region might be because of the designated uniform EF distribution therein. If EF inside the target volume can be given differently, then the result would be close to each other.

In verifying the optimization procedure effectiveness, the EF distribution in individual head models was evaluated for individually optimized montage for eight electrodes (Figure 11). A higher EF was observed in the targeted region (right accumbens) than non-targeted region for most subjects. In the majority of subjects, a high EF was distributed in the dorsolateral prefrontal cortex, the inferior frontal cortex, or the region near the longitudinal fissure, depending on the montages.

During optimization, the four electrode positions, F4, F7, F8, and Fp1, were selected in most subjects. In addition, the optimized montage for the right accumbens (Cz-F7-F8-Fp1-O1-O2-T7-T8) was the same in two subjects (Subjects 10 and 17). Considering potential clinical application, EF in the brain for group-level optimized montage was assessed to avoid optimization for different individuals. Figure 12 shows the average and relative SD of EF strength when using the same electrode pattern for all subjects and optimizing only the current ratios. For this scenario, higher EFs were identified in the target area than remaining regions. The EF strength variation in other regions, including the target tissue, was insignificantly different. Our results are similar to those reported in the study of target optimization for multichannel tDCS (Sadleir et al., 2012; Saturnino et al., 2019a).

One limitation of this study is that the accuracy of these models somehow relies on the MRI data quality and assumptions made during segmentation (Rashed et al., 2021). In addition, MR images used are based on male subjects with a specific age range, potentially limiting the generalizability of the results. The assignment of tissue conductivity, including the smooth transition of tissue conductivity without a segmentation model, can be found in our previous studies (Diao et al., 2022, 2023).

This study provided valuable insights into the computational procedure for the montage optimization of tES with different electrode numbers at individual and group levels. The main contribution focused on computational cost reduction regarding the EF surface of the target area, considering optimization in potential clinical applications at the group level as well as personalized treatment.

## Data availability statement

The raw data supporting the conclusions of this article will be made available by the authors, without undue reservation.

## Author contributions

HN: Data curation, Formal analysis, Methodology, Software, Validation, Visualization, Writing – original draft, Writing – review & editing. SK: Conceptualization, Investigation, Supervision, Writing – review & editing. NO: Conceptualization, Writing – review & editing. AH: Conceptualization, Formal analysis, Funding acquisition, Investigation, Methodology, Project administration, Supervision, Writing – original draft, Writing – review & editing.

## References

[ref1] Akhtari M. Bryant H. C. Mamelak A. N. Flynn E. R. Heller L. Shih J. J.. (2002). Conductivities of three-layer live human skull. Brain Topogr. 14, 151–167. doi: 10.1023/a:1014590923185, PMID: 12002346

[ref2] Akhtari M. Mandelkern M. Bui D. Salamon N. Vinters H. V. Mathern G. W. (2010). Variable anisotropic brain electrical conductivities in epileptogenic foci. Brain Topogr. 23, 292–300. doi: 10.1007/s10548-010-0144-z 20440549 PMC2914871

[ref3] Akhtari M. Salamon N. Duncan R. Fried I. Mathern G. W. (2006). Electrical conductivities of the freshly excised cerebral cortex in epilepsy surgery patients; correlation with pathology, seizure duration, and diffusion tensor imaging. Brain Topogr. 18, 281–290. doi: 10.1007/s10548-006-0006-x, PMID: 16858632

[ref4] Baumann S. B. Wozny D. R. Kelly S. K. Meno F. M. (1997). The electrical conductivity of human cerebrospinal fluid at body temperature. I.E.E.E. Trans. Biomed. Eng. 44, 220–223. doi: 10.1109/10.554770, PMID: 9216137

[ref5] Berton O. Nestler E. J. (2006). New approaches to antidepressant drug discovery: beyond monoamines. Nat. Rev. Neurosci. 7, 137–151. doi: 10.1038/nrn1846, PMID: 16429123

[ref6] Bikson M. Datta A. Rahman A. Scaturro J. (2010). Electrode montages for tDCS and weak transcranial electrical stimulation: role of “return” electrode’s position and size. Clin. Neurophysiol. 121, 1976–1978. doi: 10.1016/j.clinph.2010.05.020, PMID: 21035740 PMC2983105

[ref7] Bragard D. Chen A. C. N. Plaghki L. (1996). Direct isolation of ultra-late (C-fibre) evoked brain potentials by CO2 laser stimulation of tiny cutaneous surface areas in man. Neurosci. Lett. 209, 81–84. doi: 10.1016/0304-3940(96)12604-5, PMID: 8761987

[ref8] Caulfield K. A. George M. S. (2022). Optimized APPS-tDCS electrode position, size, and distance doubles the on-target stimulation magnitude in 3000 electric field models. Sci. Rep. 12:20116. doi: 10.1038/s41598-022-24618-3, PMID: 36418438 PMC9684449

[ref9] Chhatbar P. Y. Kautz S. A. Takacs I. Rowland N. C. Revuelta G. J. George M. S.. (2018). Evidence of transcranial direct current stimulation-generated electric fields at subthalamic level in human brain in vivo. Brain Stimul. 11, 727–733. doi: 10.1016/j.brs.2018.03.006, PMID: 29576498 PMC6019625

[ref10] Dale A. M. Fischl B. Sereno M. I. (1999). Cortical surface-based analysis. NeuroImage 9, 179–194. doi: 10.1006/nimg.1998.0395 9931268

[ref11] Datta A. Bansal V. Diaz J. Patel J. Reato D. Bikson M. (2009). Gyri-precise head model of transcranial direct current stimulation: improved spatial focality using a ring electrode versus conventional rectangular pad. Brain Stimul. 2, 201–207.e1. doi: 10.1016/j.brs.2009.03.005, PMID: 20648973 PMC2790295

[ref12] Dawson T. W. Stuchly M. A. (1998). High-resolution organ dosimetry for human exposure to low-frequency magnetic fields. IEEE Trans. Magn. 34, 708–718. doi: 10.1109/20.668071

[ref13] Diao Y. Liu L. Deng N. Lyu S. Hirata A. (2023). Tensor-conductance model for reducing the computational artifact in target tissue for low-frequency dosimetry. Phys. Med. Biol. 68:205014. doi: 10.1088/1361-6560/acfae0, PMID: 37722382

[ref14] Diao Y. Rashed E. A. Hirata A. (2022). Induced electric field in learning-based head models with smooth conductivity for exposure to uniform low-frequency magnetic fields. IEEE Trans. Electromagn. Compat. 64, 1969–1977. doi: 10.1109/TEMC.2022.3212860

[ref15] Dichiara G. Bassareo V. (2007). Reward system and addiction: what dopamine does and doesn’t do. Curr. Opin. Pharmacol. 7, 69–76. doi: 10.1016/j.coph.2006.11.003, PMID: 17174602

[ref16] Edwards D. Cortes M. Datta A. Minhas P. Wassermann E. M. Bikson M. (2013). Physiological and modeling evidence for focal transcranial electrical brain stimulation in humans: a basis for high-definition tDCS. NeuroImage 74, 266–275. doi: 10.1016/j.neuroimage.2013.01.042, PMID: 23370061 PMC4359173

[ref17] Ekhtiari H. Tavakoli H. Addolorato G. Baeken C. Bonci A. Campanella S.. (2019). Transcranial electrical and magnetic stimulation (tES and TMS) for addiction medicine: a consensus paper on the present state of the science and the road ahead. Neurosci. Biobehav. Rev. 104, 118–140. doi: 10.1016/j.neubiorev.2019.06.007, PMID: 31271802 PMC7293143

[ref18] Ferrucci R. Cortese F. Priori A. (2015). Cerebellar tDCS: how to do it. Cerebellum 14, 27–30. doi: 10.1007/s12311-014-0599-7, PMID: 25231432 PMC4318979

[ref19] Freygang W. H. Jr. Landau W. M. (1955). Some relations between resistivity and electrical activity in the cerebral cortex of the cat. J. Cell. Comp. Physiol. 45, 377–392. doi: 10.1002/jcp.1030450305, PMID: 13263358

[ref20] Gabriel S. Lau R. W. Gabriel C. (1996). The dielectric properties of biological tissues: II. Measurements in the frequency range 10 Hz to 20 GHz. Phys. Med. Biol. 41, 2251–2269. doi: 10.1088/0031-9155/41/11/002, PMID: 8938025

[ref21] Gabriel C. Peyman A. Grant E. H. (2009). Electrical conductivity of tissue at frequencies below 1 MHz. Phys. Med. Biol. 54, 4863–4878. doi: 10.1088/0031-9155/54/16/002, PMID: 19636081

[ref22] Gomez-Tames J. Asai A. Hirata A. (2019a). Significant group-level hotspots found in deep brain regions during transcranial direct current stimulation (tDCS): a computational analysis of electric fields. Clin. Neurophysiol. 131, 755–765. doi: 10.1016/j.clinph.2019.11.018, PMID: 31839398

[ref23] Gomez-Tames J. Asai A. Mikkonen M. Laakso I. Tanaka S. Uehara S.. (2019b). Group-level and functional-region analysis of electric-field shape during cerebellar transcranial direct current stimulation with different electrode montages. J. Neural Eng. 16:036001. doi: 10.1088/1741-2552/ab0ac5, PMID: 30808008

[ref24] Gomez-Tames J. Kutsuna T. Tamura M. Muragaki Y. Hirata A. (2018). Intraoperative direct subcortical stimulation: comparison of monopolar and bipolar stimulation. Phys. Med. Biol. 63:225013. doi: 10.1088/1361-6560/aaea06, PMID: 30418938

[ref25] Gomez-Tames J. Laakso I. Haba Y. Hirata A. Poljak D. Yamazaki K. (2017). Computational artifacts of the in situ electric field in anatomical models exposed to low-frequency magnetic field. IEEE Trans. Electromagn. Compat. 60, 589–597. doi: 10.1109/TEMC.2017.2748219

[ref26] Gomez-Tames J. Sugiyama Y. Laakso I. Tanaka S. Koyama S. Sadato N.. (2016). Effect of microscopic modeling of skin in electrical and thermal analysis of transcranial direct current stimulation. Phys. Med. Biol. 61, 8825–8838. doi: 10.1088/1361-6560/61/24/8825, PMID: 27897150

[ref27] Goodwill A. M. Lum J. A. G. Hendy A. M. Muthalib M. Johnson L. Albein-Urios N.. (2017). Using non-invasive transcranial stimulation to improve motor and cognitive function in Parkinson’s disease: a systematic review and meta-analysis. Sci. Rep. 7:14840. doi: 10.1038/s41598-017-13260-z, PMID: 29093455 PMC5665996

[ref28] Guler S. Dannhauer M. Erem B. Macleod R. Tucker D. Turovets S.. (2016). Optimization of focality and direction in dense electrode array transcranial direct current stimulation (tDCS). J. Neural Eng. 13:036020. doi: 10.1088/1741-2560/13/3/036020, PMID: 27152752 PMC5198846

[ref29] Huang Y. Liu A. A. Lafon B. Friedman D. Dayan M. Wang X.. (2017). Measurements and models of electric fields in the in vivo human brain during transcranial electric stimulation. eLife 6:e18834. doi: 10.7554/eLife.18834, PMID: 28169833 PMC5370189

[ref30] Huff W. Lenartz D. Schormann M. Lee S.-H. Kuhn J. Koulousakis A.. (2010). Unilateral deep brain stimulation of the nucleus accumbens in patients with treatment-resistant obsessive-compulsive disorder: outcomes after one year. Clin. Neurol. Neurosurg. 112, 137–143. doi: 10.1016/j.clineuro.2009.11.006, PMID: 20006424

[ref31] Khadka N. Bikson M. (2020). Role of skin tissue layers and ultra-structure in transcutaneous electrical stimulation including tDCS. Phys. Med. Biol. 65:225018. doi: 10.1088/1361-6560/abb7c1, PMID: 32916670

[ref32] Khan A. Antonakakis M. Vogenauer N. Haueisen J. Wolters C. H. (2022). Individually optimized multi-channel tDCS for targeting somatosensory cortex. Clin. Neurophysiol. 134, 9–26. doi: 10.1016/j.clinph.2021.10.016, PMID: 34923283

[ref33] Kobayashi M. Pascual-Leone A. (2003). Transcranial magnetic stimulation in neurology. Lancet Neurol. 2, 145–156. doi: 10.1016/S1474-4422(03)00321-1 12849236

[ref34] Laakso I. Hirata A. (2012). Fast multigrid-based computation of the induced electric field for transcranial magnetic stimulation. Phys. Med. Biol. 57, 7753–7765. doi: 10.1088/0031-9155/57/23/7753, PMID: 23128377

[ref35] Laakso I. Mikkonen M. Koyama S. Hirata A. Tanaka S. (2019). Can electric fields explain inter-individual variability in transcranial direct current stimulation of the motor cortex? Sci. Rep. 9:626. doi: 10.1038/s41598-018-37226-x, PMID: 30679770 PMC6345748

[ref36] Laakso I. Tanaka S. Koyama S. De Santis V. Hirata A. (2015). Inter-subject variability in electric fields of motor cortical tDCS. Brain Stimul. 8, 906–913. doi: 10.1016/j.brs.2015.05.002, PMID: 26026283

[ref37] Langevin J. P. Chen J. W. Y. Koek R. J. Sultzer D. L. Mandelkern M. A. Schwartz H. N.. (2016). Deep brain stimulation of the basolateral amygdala: targeting technique and electrodiagnostic findings. Brain Sci. 6:8. doi: 10.3390/brainsci6030028, PMID: 27517963 PMC5039457

[ref38] Latikka J. Kuurne T. Eskola H. (2001). Conductivity of living intracranial tissues. Phys. Med. Biol. 46, 1611–1616. doi: 10.1088/0031-9155/46/6/302, PMID: 11419622

[ref39] Lee S. Park J. Lee C. Ahn J. Ryu J. Lee S.-H.. (2023). Determination of optimal injection current pattern for multichannel transcranial electrical stimulation without individual MRI using multiple head models. Comput. Methods Prog. Biomed. 243:107878. doi: 10.1016/j.cmpb.2023.107878, PMID: 37890288

[ref40] Lindenblatt G. Silny J. (2001). A model of the electrical volume conductor in the region of the eye in the ELF range. Phys. Med. Biol. 46, 3051–3059. doi: 10.1088/0031-9155/46/11/319, PMID: 11720363

[ref9001] Levkovitz Y. Harel E. V. Roth Y. Braw Y. Most D. Katz L. N.. (2009). Deep transcranial magnetic stimulation over the prefrontal cortex: Evaluation of antidepressant and cognitive effects in depressive patients. Brain Stimul. 2, 188–200. doi: 10.1016/j.brs.2009.08.002, PMID: 20633419

[ref41] Mikkonen M. Laakso I. Tanaka S. Hirata A. (2020). Cost of focality in TDCS: Interindividual variability in electric fields. Brain Stimul. 13, 117–124. doi: 10.1016/j.brs.2019.09.017, PMID: 31606449

[ref42] Montgomery E. B. Huang H. Walker H. C. Guthrie B. L. Watts R. L. (2011). High-frequency deep brain stimulation of the putamen improves bradykinesia in Parkinson’s disease. Mov. Disord. 26, 2232–2238. doi: 10.1002/mds.23842, PMID: 21714010 PMC4151533

[ref43] Nitsche M. A. Boggio P. S. Fregni F. Pascual-Leone A. (2009). Treatment of depression with transcranial direct current stimulation (tDCS): a review. Exp. Neurol. 219, 14–19. doi: 10.1016/j.expneurol.2009.03.038 19348793

[ref44] Opitz A. Paulus W. Will S. Antunes A. Thielscher A. (2015). Determinants of the electric field during transcranial direct current stimulation. NeuroImage 109, 140–150. doi: 10.1016/j.neuroimage.2015.01.033 25613437

[ref45] Palm U. Hasan A. Strube W. Padberg F. (2016). tDCS for the treatment of depression: a comprehensive review. Eur. Arch. Psychiatry Clin. Neurosci. 266, 681–694. doi: 10.1007/s00406-016-0674-9, PMID: 26842422

[ref46] Ranck J. B. (1963). Specific impedance of rabbit cerebral cortex. Exp. Neurol. 7, 144–152. doi: 10.1016/S0014-4886(63)80005-9, PMID: 13990734

[ref47] Rashed E. A. Gomez-Tames J. Hirata A. (2021). Influence of segmentation accuracy in structural MR head scans on electric field computation for TMS and tES. Phys. Med. Biol. 66:064002. doi: 10.1088/1361-6560/abe223, PMID: 33524957

[ref48] Reilly J. P. Hirata A. (2016). Low-frequency electrical dosimetry: research agenda of the IEEE international committee on electromagnetic safety. Phys. Med. Biol. 61, R138–R149. doi: 10.1088/0031-9155/61/12/R138, PMID: 27223463

[ref49] Rodman A. M. Milad M. R. Deckersbach T. Im J. Chou T. Dougherty D. D. (2012). Neuroimaging contributions to novel surgical treatments for intractable obsessive–compulsive disorder. Expert. Rev. Neurother. 12, 219–227. doi: 10.1586/ern.11.189, PMID: 22288677

[ref50] Sadleir R. J. Vannorsdall T. D. Schretlen D. J. Gordon B. (2012). Target optimization in transcranial direct current stimulation. Front. Psych. 3, 1–13. doi: 10.3389/fpsyt.2012.00090, PMID: 23087654 PMC3474130

[ref51] Sagliano L. Atripaldi D. De Vita D. D’Olimpio F. Trojano L. (2019). Non-invasive brain stimulation in generalized anxiety disorder: a systematic review. Prog. Neuropsychopharmacol. Biol. Psychiatry 93, 31–38. doi: 10.1016/j.pnpbp.2019.03.002, PMID: 30876986

[ref52] Salvador R. Biagi M. C. Puonti O. Splittgerber M. Moliadze V. Siniatchkin M.. (2021). “Personalization of multi-electrode setups in tCS/tES: methods and advantages,” In Makarov S.N. Noetscher G.M. Nummenmaa A. (Eds.) Brain and Human Body Modeling 2020 (Cham: Springer International Publishing), 119–135. 32966022

[ref53] Saturnino G. B. Madsen K. H. Thielscher A. (2021). Optimizing the electric field strength in multiple targets for multichannel transcranial electric stimulation. J. Neural Eng. 18:014001. doi: 10.1088/1741-2552/abca15, PMID: 33181504 PMC8488191

[ref54] Saturnino G. B. Siebner H. R. Thielscher A. Madsen K. H. (2019a). Accessibility of cortical regions to focal TES: dependence on spatial position, safety, and practical constraints. NeuroImage 203:116183. doi: 10.1016/j.neuroimage.2019.116183, PMID: 31525498

[ref55] Saturnino G. B. Thielscher A. Madsen K. H. Knösche T. R. Weise K. (2019b). A principled approach to conductivity uncertainty analysis in electric field calculations. NeuroImage 188, 821–834. doi: 10.1016/j.neuroimage.2018.12.053, PMID: 30594684

[ref56] Sauvaget A. Trojak B. Bulteau S. Jiménez-Murcia S. Fernández-Aranda F. Wolz I.. (2015). Transcranial direct current stimulation (tDCS) in behavioral and food addiction: a systematic review of efficacy, technical, and methodological issues. Front. Neurosci. 9:349. doi: 10.3389/fnins.2015.00349, PMID: 26500478 PMC4598576

[ref57] Stagg C. J. Nitsche M. A. (2011). Physiological basis of transcranial direct current stimulation. Neurosci. 17, 37–53. doi: 10.1177/1073858410386614 21343407

[ref58] Stoupis D. Samaras T. (2022). Non-invasive stimulation with temporal interference: optimization of the electric field deep in the brain with the use of a genetic algorithm. J. Neural Eng. 19:056018. doi: 10.1088/1741-2552/ac89b3, PMID: 35970146

[ref59] Stoy R. D. Foster K. R. Schwan H. P. (1982). Dielectric properties of mammalian tissues from 0.1 to 100 MHz; a summary of recent data. Phys. Med. Biol. 27, 501–513. doi: 10.1088/0031-9155/27/4/002, PMID: 7089048

[ref60] Tavakoli A. V. Yun K. (2017). Transcranial alternating current stimulation (tACS) mechanisms and protocols. Front. Cell. Neurosci. 11:214. doi: 10.3389/fncel.2017.00214, PMID: 28928634 PMC5591642

[ref61] Tay G. Chilbert M. Battocletti J. Sances A. Swiontek T. Kurakami C. (1989). Measurement of Magnetically Induced Current Density in Saline In vivo. In Images of the Twenty-First Century. Proceedings of the Annual International Engineering in Medicine and Biology Society (IEEE), 1167–1168.

[ref62] Vöröslakos M. Takeuchi Y. Brinyiczki K. Zombori T. Oliva A. Fernández-Ruiz A.. (2018). Direct effects of transcranial electric stimulation on brain circuits in rats and humans. Nat. Commun. 9:483. doi: 10.1038/s41467-018-02928-3, PMID: 29396478 PMC5797140

